# 

**Published:** 1900-07-21

**Authors:** 


					ma ayjariTAij. ?? 1 ne standard or hignest purity."^-Lancet. July 21st, 1900
CADBURY'S ff ^ CADBURY'S )
ftflfiAA \m. JMc? - r.nrn a v
cocoa L cocoa
ABSOLUTELY PURE. ' W NO DRUGS OR ALKALIES USED.
ySL/Kscorv Western Inf/rmak
No. 721./Vol. XXVIII. [ffiS&g Saturday,
July 21, 1900.
^Subscnpfaon Tjerms, J pRICE TWOPENCE.

				

